# Development of an Internet-based Product-related Child Injury Textual Data Platform (IPCITDP) in China

**DOI:** 10.7189/jogh.14.04174

**Published:** 2024-08-23

**Authors:** Wangxin Xiao, Peixia Cheng, David C Schwebel, Lei Yang, Min Zhao, Shuying Zhao, Guoqing Hu

**Affiliations:** 1 Department of Epidemiology and Health Statistics; Hunan Provincial Key Laboratory of Clinical Epidemiology, Xiangya School of Public Health, Central South University, Changsha, China; 2 Department of Child, Adolescent and Women's Health, School of Public Health, Capital Medical University, Beijing, China; 3 Department of Psychology, University of Alabama at Birmingham, Birmingham, Alabama, USA; 4 National Clinical Research Center for Geriatric Disorders, Xiangya Hospital, Central South University, Changsha, China

## Abstract

**Background:**

Internet-based media stories provide valuable information for emerging risks of product-related child injury prevention and control, but critical methodological challenges and high costs of data acquisition and processing restrict practical use by stakeholders.

**Methods:**

We constructed a data platform through literature reviews and multi-round research group discussions. Developed components included standard search strategies, filtering criteria, textual document classification, information extraction standards and a keyword dictionary. We used ten thousand manually labelled media stories to validate the textual document classification model, which was established using the Bidirectional Encoder Representation from Transformers (BERT). Multiple information extraction methods based on natural language processing algorithms were adopted to extract data for 29 structured variables from media stories. They were evaluated through manual validation of 1000 media stories about product-related child injury. We mapped the geographic distribution of media sources and media-reported product-related child injury events.

**Results:**

We developed an internet-based product-related child injury textual data platform, IPCITDP, consisting of four layers – automatic data search and acquisition, data processing, data storage, and data application – concerning product-related child injury online media stories in China. Each layer occurred daily. External validation demonstrated high performance for the BERT classification model we established (accuracy = 0.9703) and the combined information extraction strategies (accuracy >0.70 for 25 variables). As of 31 December 2023, IPCITDP collected 35 275 eligible product-related child injury reports from 13 261 news media websites or social media platform accounts which were geographically located across all 31 mainland Chinese provinces and covered over 97% of prefecture-level cities. The injury cases in IPCITDP were typically reported several months or years earlier than official announcements about the product-related child injury risks. Our data platform added data concerning 15 supplementary variables that the national product-related injury surveillance system lacks. Two examples demonstrate the value of IPCITDP in supplementing existing data and providing early epidemiological detection of emerging signals concerning product-related child injury: magnetic beads and electric self-balancing scooters.

**Conclusions:**

Our data platform provides injury data that can support early detection of new product-related child injury characteristics and supplement existing data sources to reduce the burden of product-related injury among Chinese children.

A product can be defined as any article produced by manufacturers and used by consumers in or around specific places such as the home, school, or recreational areas [1]. Products comprise an important etiological factor for child injury, especially when those products are defective or improperly used [2,3]. According to The World Report on Child Injury Prevention [4], children frequently experience product-related injury. In the USA, product-related injuries caused approximately 4.25 million emergency department visits, 151 682 hospitalisations, and 1370 deaths among children aged 0–19 years old in 2022 [5].

The prevention of product-related child injuries is embedded in target 3.2 of the Sustainable Development Goals (SDGs), ‘ending preventable deaths of newborns and under-five children by 2030’ [6]. The recently released National Child Development Outline of China, 2021–2030, issued by the state council of China, highlights recommended response strategies and measures to reduce and prevent product-related child injuries. These recommendations include, ‘establish and improve mandatory standards and legislation for children’s products to strengthen product quality and safety, encourage consumers to legally identify and report product defects, and further reinforce information monitoring and defective product recalls relevant to product-related child injury’ [7].

High-quality epidemiological data form the base of research and policy-making concerning major public health challenges, including product-related child injuries [8], and are essential to meet government recommendations to monitor trends in product defects that may cause child injury. So far, only a few countries and regions have established national injury surveillance systems to collect product-related injury data, including the USA [5], UK [9], Australia [10], Europe [11], and China [12]. These surveillance systems provide valuable public health data, but they rely entirely on predefined structured variables and thus are insensitive to newly emerging product-related injury patterns such as child injuries related to new products like magnetic beads, electric balance scooters, or shared bikes, or to changing trends in improper installation, use or storage of products [13,14]. Further, existing surveillance systems typically require several months or years to collect, clean, and release data, leading to delays in using the findings to detect and address new or emerging epidemiological characteristics in a timely manner [15,16]. Last, only some details of the surveillance data are shared publicly in countries like China, somewhat restricting their utility in product-related child injury research and prevention practice [17].

Rapidly-growing multi-source media stories online, including social media stories, offer a valuable, timely, and free data source to supplement existing systems and monitor product-related child injury trends [18–20]. As of 30 September 2023, the Cyberspace Administration of China approved 3523 internet news and information service units to operate internet websites, WeChat public accounts, applications, blogs, micro-blogs, forums, instant messaging tools, live media, and other service items [21]. Together, these media release extensive daily media stories, some of which involve product-related child injury events.

Given the absence of data sources to support early detection of newly emerging product-related child injury trends and characteristics in China, the present project was designed to evaluate the research question of whether an automatic data platform could be developed to regularly and systematically collect media reports concerning child product-related injury and transfer them into structured and usable data. Driven by injury prevention theory, we used natural language processing, machine learning, and computer programming to develop a platform, the Internet-based Product-related Child Injury Textual Data Platform (IPCITDP), that automatically collects and processes internet-based media stories in Chinese concerning product-related child injury, as well as storing and visualising the structured injury data for use by interested stakeholders. We evaluated the IPCITDP data outcomes through multiple strategies.

## METHODS

**Figure 1:**
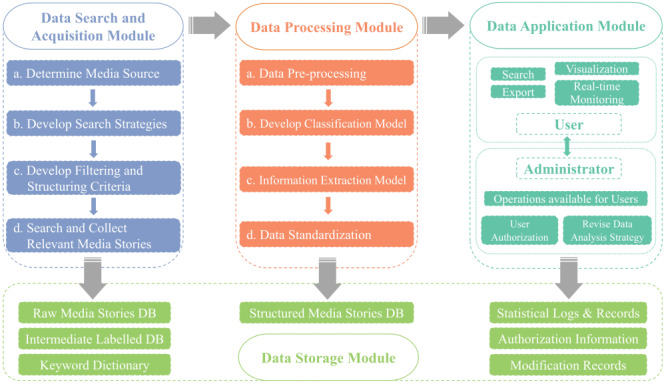
System architecture of the Internet-based Product-related Child Injury Textual Data Platform (IPCITDP). DB – database.

The IPCITDP platform we developed includes four modules: data search and acquisition, data processing, data storage, and data application (**Figure 1**). Below, we briefly describe key methods used to develop each module.

### Module one: Data search and acquisition

#### Determining media source

Through preliminary literature reviews and multi-round group discussions, we selected online news media websites and social media platforms as the two data sources for media stories. We included 20 national news media websites and 31 provincial news media websites according to the following criteria: (1) PageRank value [22] is greater than four; (2) reported injury events occurred in mainland China and the media stories were written in Chinese; and (3) the media story covered a comprehensive and true story rather than considering fictional stories (e.g. product-related injuries in games or movies) or focusing only on a specific topic such as educational or legal matters about an incident but not containing the epidemiological details of injury event.

We included social media platforms owned by the government and by social organisations, as well as individual social media user accounts operated by Sina Micro-blog and Tencent’s WeChat, the two most influential and popular social media platforms in China. All social media user accounts on Sina Micro-blog and Tencent’s WeChat were required to operate legally, be registered through certification of identification cards or institutional registration certificates [23,24], and publish media stories that strictly complied to the Cybersecurity Law of the People’s Republic of China [25]. All information published on Sina Micro-blog and Tencent’s WeChat is reviewed by the government and illegal, false, and private information is eliminated. Our data omits private or sensitive information.

#### Developing criteria for media stories searching and text-processing

Based on relevant literature [3,26–28] and multi-round focus group discussions, we established search strategies and filtering criteria for product-related child injury media stories, as detailed in Textbox S1 in the **Online Supplementary Document**. Based on etiological factors described in the Haddon matrix [3], external cause of injury listings in the 10th International Classification of Disease (ICD-10) [26], the national consumer product normative classification of China [27], and the Product Quality Law of China [28], we developed a list of 29 structured variables to extract key information from media stories. These variables consisted of eight basic variables such as date, occurring location and geographic place where the injury event occurred; six product-related variables involving name, type, and other characteristics of the product; nine human factors concerning the children and their supervisors; and six variables concerning environmental characteristics and the external cause of injury. Detailed descriptions of the 29 structured variables appear in Table S1 in the **Online Supplementary Document**. Some of the 29 variables comprise information that the national product-related injury surveillance system does not collect, offering supplemental data to existing data sources such as environmental information concerning where product-related injuries occur and information about adult supervisors of children who experience a product-related injury.

To support searching and text-processing of media stories, we developed a keyword dictionary with six chapters: child-related, product-related, injury-related, environment-related, activity-related, and other keywords.

#### Searching potentially relevant media stories

According to the Cyber Security Law of the People’s Republic of China [25], we formulated the focused web crawler algorithm based on the established search strategies described above and used the algorithm to automatically collect potentially relevant raw media stories every 24 hours. During the algorithm search, we gathered the article title, publication date and time, and the article’s main text, as well as the name of news website or platform account, and the uniform resource locator (URL) link of the news website, Tencent WeChat, or Sina Micro-blog platform that published the news story about product-related child injuries.

We limited the time period of our search to those stories appearing in 2010 or later because potentially eligible media stories that were published prior to 2010 are no longer consistently available online due to the cost of storing the large number of media stories [29].

### Module two: Processing the textual media stories

#### Developing the classification model for media stories

All searched media stories used for fitting the classification model (including training data set, validation data set, and test validation data set) were first pre-processed via natural language models and manual cleaning.

Next, we installed the pre-training model for Chinese text analysis using massive unsupervised corpus with word piece masking from GitHub. Based on our preliminary analysis and prior research [29], we chose the Bidirectional Encoder Representation from Transformers (BERT), which was created based on multi-layer and bidirectional training transformers encoder by Google AI team in 2018 [30], to establish the classification model used to identify eligible product-related child injury media stories.

Last, we randomly selected 10 000 potentially relevant media stories and organised a group of four researchers to manually and independently classify them into two categories, eligible report or ineligible report. All researchers received extensive training prior to their coding and they conducted classification based on a written operational manual (Textbox S1 in the **Online Supplementary Document**). Re-evaluation of 2000 from the labelled 10 000 media stories (validation sample) by another researcher showed high consistency (99.78%). We then divided the 10 000 labelled media stories into three data sets – a training data set (8000 reports), a validation data set (1000 reports), and a test data set (1000 reports). Detailed parameters of the classification model development are presented in Table S2 in the **Online Supplementary Document**. Following previous research [29], we calculated the model performance metrics, including accuracy, precision, recall/sensitivity, specificity, false positive rate and *F*_1_ score. The development of the classification model was performed through Python, version 3.7 (Python Software Foundation, Wilmington, DE, USA) and TensorFlow, version 2.2 (Google Brain, San Francisco, CA, USA) [31].

#### Extracting information about 29 structured variables

Once eligible media stories were identified, we flexibly adopted multiple natural language processing algorithms containing regular expression, named entity recognition, keyword matching and dependency syntactic parsing to extract information concerning the 29 predefined structured variables (**Table 1**). The algorithm used several different strategies to extract information about each of the 29 variables for each news story: (1) regular expression was used to extract information concerning numerical variables from media stories based on specific syntactic rules; (2) named entity recognition was applied to identify specific entity names in the media stories including date, geographic names, people’s names, specialised names, etc.; and (3) keyword matching and dependency syntactic parsing were employed to match specific keywords from media stories and then detect interdependence between different words.

**Table 1:** Methods for extracting information of 29 related variables and their performances compared to manual evaluations

Variable	Data extraction method being used			Media stories including the variable	Extraction rate (%)*	Accuracy (%)†
	**Regular expression**	**Named entity recognition**	**Keyword matching and dependency syntactic parsing**
**Basic characteristics of injury event**						
1. Date the injury event occurred	√	√	√	800	99.26	90.65
2. Geographic location where the injury event occurred		√	√	808	98.54	88.60
3. Type of place where the injury event occurred			√	811	99.75	80.52
4. Nature (type) of injury			√	837	99.52	85.07
5. Body part injured			√	826	99.52	80.75
6. External cause of injury			√	810	99.75	79.75
7. Clinical diagnosis of injury	√		√	174	19.68	44.94
8. Injury outcome						
*8.1 Number of deaths*	√		√	412	50.80	78.81
*8.2 Number of injured persons*	√		√	547	66.46	83.96
*8.3 Number of child deaths*	√		√	381	46.98	81.41
*8.4 Number of injured children*	√		√	554	66.99	92.02
**Product-related variable**						
9. Number of involved products			√	818	98.44	83.41
10. Product name			√	825	98.92	79.11
11. Brand name of product			√	-‡	-‡	-‡
12. Characteristics of product			√	27	3.23	62.77
13. Type of product			√	829	99.16	70.76
14. Disposition of injury			√	811	99.51	84.71
**Children and supervisor-related variable**						
15. Number of involved children	√			800	99.38	78.25
16. Age of injured child	√			665	81.80	87.52
17. Sex of injured child			√	793	99.50	84.11
18. Activity of child(ren) as injury happened			√	827	99.52	76.90
19. Number of supervisors	√			207	24.76	70.14
20. Age of supervisor	√			98	12.34	87.33
21. Sex of supervisor			√	817	99.51	77.11
22. Supervisor of the injured child			√	644	78.54	73.77
23. Whether the supervisor was physically with the child when the injury event occurred			√	829	99.40	83.72
**Other variables related to the injury environment and causal factors**						
24. Etiological factors leading to injury						
*24.1 Human-related etiological factor*			√	834	96.64	86.50
*24.2 Product-related etiological factor*			√	740	86.25	89.94
*24.3 Description of the detailed etiological factor*			√	698	77.64	4.81
25. Weather conditions			√	-‡	-‡	-‡
26. Air quality			√	-‡	-‡	-‡
27. Temperature			√	-‡	-‡	-‡
28. Other adverse environmental conditions	√		√	-‡	-‡	-‡
29. Product-related preventive measure	√		√	377	48.33	36.73

*The proportion of media stories including the information of the variable being extracted.

†The consistency of extracted databased on data extraction models compared to manual extraction strategies.

‡Relevant results were not shown due to inadequate media stories including the information.

To validate the natural language processing algorithms, we recruited eight researchers to manually extract information concerning the 29 variables for 1000 randomly selected media stories. All eight researchers received extensive training prior to extracting the information and used a written operational manual consisting of case definitions and processing operations for each of the 29 variables to conduct their extraction (Table S2 in the **Online Supplementary Document**). Using the manual evaluations as a standard, we calculated the extraction rate and accuracy of information extraction algorithms for the 1000 media stories on the 29 variables. Algorithm-based information extraction was performed using Python (version 3.7) and the Stanford Core NLP Toolkit [32].

### Module three: Data storage

**Figure 2:**
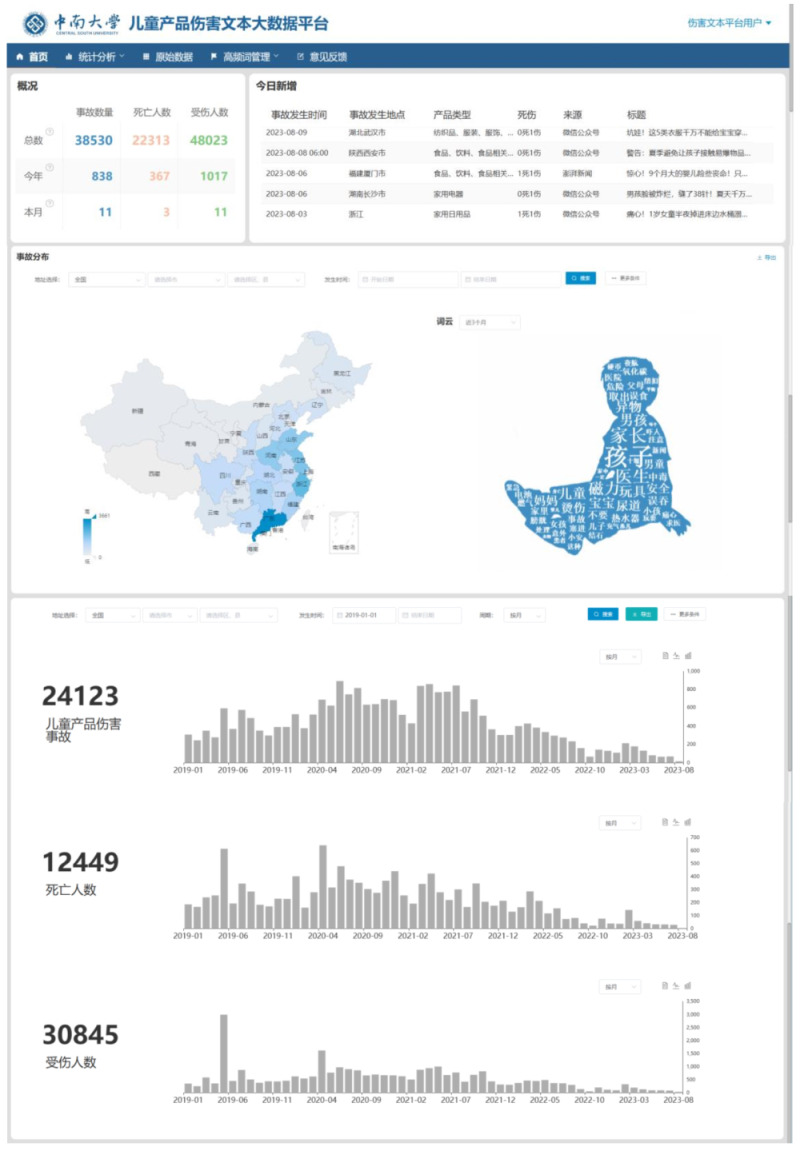
Illustration of the Internet-based Product-related Child Injury Textual Data Platform (IPCITDP).

We stored all relevant data in Alibaba Cloud Relational Database Service (RDS) for MySQL (version 5.6), a widely used structured query language (SQL) database [33]. Five kinds of data were automatically collected, processed, and stored by IPCITDP every 24 hours: (1) raw media stories; (2) intermediate labelled data from information extraction, referring to unstructured words, phrases, or sentences; (3) the keyword dictionary we developed; (4) textual features related to the 29 structured variables; and (5) statistical logs and records of ordinary users and platform administrators. **Figure 2** displays the main interface of IPCITDP.

### Module four: Data application

The IPCITDP principally serves two groups, ordinary users and platform administrators. Ordinary users can perform the following operations: (1) selectively search, view, download, and export structured data; and (2) customise personalised data visualisation, including bar charts, pie charts, line graphs, word clouds and geographical maps.

Platform administrators are powered to perform additional operations, including: (1) selectively search, view, download, and export all data in the platform; (2) adjust and update the classification models and data extraction algorithms; (3) improve the configuration and allocation of storage resources; and (4) manage user authorisation and operation permissions.

### Performance evaluation of IPCITDP

We evaluated four attributes of IPCITDP: (1) accuracy of the media story classification model and information extraction algorithms compared to manual evaluations by researchers; (2) geographic coverage of media sources and product-related child injury events indexed by the IPCITDP; (3) provision of supplemental data that extend beyond existing databases; and (4) early detection of new product-related child injury characteristics.

## RESULTS

After development, by 31 December 2023, the IPCITDP platform had collected 35 275 eligible media stories about product-related child injury since 1 January 2010. We report the major results below.

### Accuracy of classification model and information extraction algorithms

Compared to manual evaluations, the BERT classification model displayed high accuracy for both the test data set (precision = 0.9355, accuracy = 0.9703, *F*_1_ score = 0.9700) and the validation data set (precision = 0.9235, accuracy = 0.9680, *F*_1_ score = 0.9679) (**Table 2**).

**Table 2:** Performance evaluation of BERT classification model

Performance indicator	Value (%)
Validation data set	
*Accuracy*	96.80
*Precision of average*	95.07
*Precision of positive*	92.35
*Recall*	96.80
*F_1_ score*	96.79
Test data set	
*Accuracy*	97.03
*Precision of average*	95.64
*Precision of positive*	93.55
*Recall*	97.03
*F_1_ score*	97.00

BERT – Bidirectional Encoder Representations from Transformers

The information extraction rate was over 95% for 16 variables and ranged from 50.80 to 86.25% for 7 variables, but was less than 50% for 6 included variables. Information extraction rates for 5 variables were not calculated because the 1000 selected media stories did not include information about those variables. Two variables (8, injury outcome; and 24, etiological factor of injury) were divided respectively into four and three sub-variables to collect detailed information (**Table 1**).

With manual extraction as the gold standard, the accuracy of the information extraction algorithms exceeded 70% for 25 variables. The four exceptions were accuracy of 62.77% for characteristic of the product (variable 12), 44.94% for clinical diagnosis of injury (variable 7), 36.73% for product-related preventive measure (variable 29), and 4.81% for description of the detailed etiological factor (sub-variable 24.3) (**Table 1**).

### Geographic coverage

**Figure 3:**
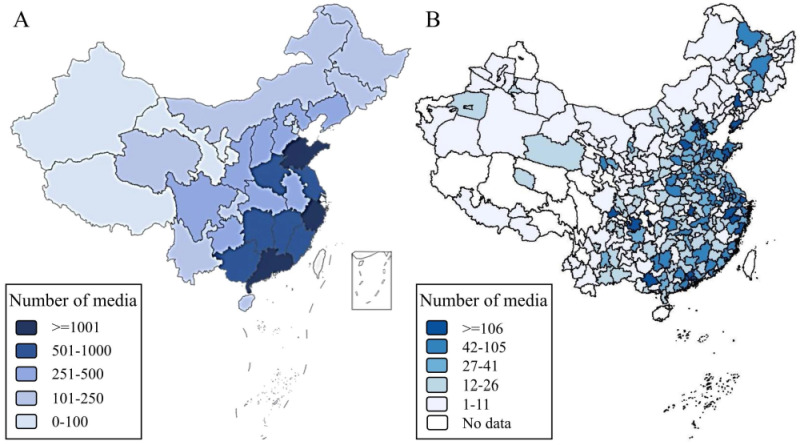
Geographic coverage of IPCITDP-collected news media websites or social media accounts reporting product-related child injury in mainland China, 2010–2023. **Panel A.** Province level. **Panel B.** Prefecture level. The IPCITDP collected media stories from the 31 provinces in mainland China, and excluded those from Hong Kong, Macao, and Taiwan due to different regulations for product-related child injury in those regions. IPCITDP – Internet-based Product-related Child Injury Textual Data Platform.

The IPCITDP collected 35 275 media stories concerning product-related child injury events that occurred between 2010 and 2023; these reports were published by 13 261 media sources (news media websites or social media platform accounts) from all 31 Chinese provinces (100%) and 311 of the 333 prefecture-level administrative divisions (93.39%) in mainland China (**Figure 3**, panels A–B). Media reports about product-related child injuries were most common in the heavily-populated provinces of Guangdong (n = 1791), Zhejiang (n = 1082), and Shandong (n = 1046), and less common in the sparsely-populated provinces of Tibet (n = 8) and Gansu (n = 78). The three most common media sources that published product-related child injury media stories were 360 Fast News (n = 5206), Sina News (n = 1316), and China News (n = 430) (Figure S1 in the **Online Supplementary Document**).

**Figure 4:**
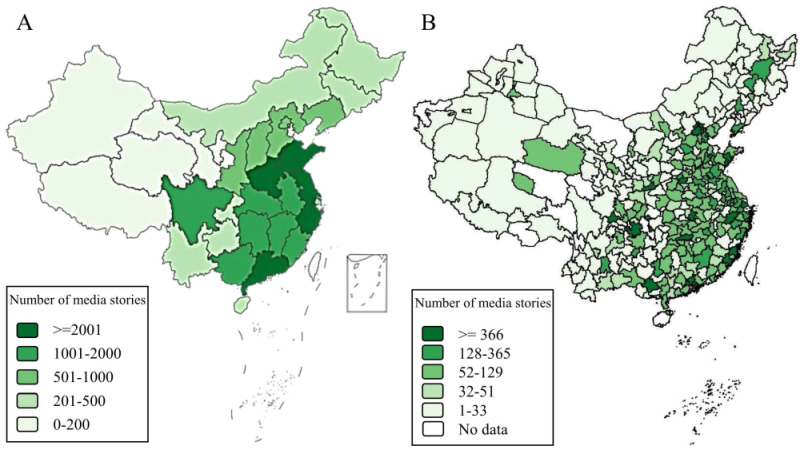
Geographic distribution of IPCITDP-collected product-related child injury media stories in mainland China, 2010–2023. **Panel A.** Province level. **Panel B.** Prefecture level. The IPCITDP collected product-related child injury from media stories of 31 provinces in mainland China, and excluded those from Hong Kong, Macao and Taiwan due to different regulations for product-related child injury. IPCITDP – Internet-based Product-related Child Injury Textual Data Platform.

Of the 35 275 collected product-related child injury media stories, 30 538 (86.57%) specified the geographic location where the injuries occurred. These locations covered all 31 mainland China provinces and 324 of the 333 prefecture-level administrative divisions (97.30%) (**Figure 4**, panels A–B). Media-reported product-related child injuries were most common in Guangdong (n = 4074), Zhejiang (n = 2730), and Jiangsu (n = 2278), and least common in Tibet (n = 14) and Qinghai (n = 166).

### Provision of data to supplement existing sources

**Figure 5:**
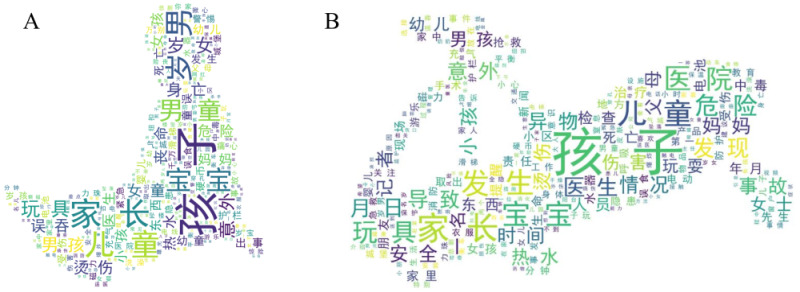
Frequency distribution of keywords based on 35 275 platform-collected media stories concerning product-related child injury between 2010 and 2023. **Panel A.** Word cloud based on article titles. **Panel B.** Word cloud based on the main body of media stories. Only the 100 most frequently cited keywords are displayed. Larger Chinese characters indicate that these keywords were more frequently mentioned in the media stories.

The IPCITDP collects data concerning 15 variables not available in the National Injury Surveillance System (NISS) of China [34], including two basic variables (geographic location where the injury event occurred, injury outcome), one product-related variable (characteristics of product), six child-related or supervisor-related variables (number of involved children, number of supervisors, age and sex of supervisor, supervisor of the injured child, and supervision behaviour when the injury event occurs), and six other variables (human-related aetiological factor, four types of environmental conditions, and product-related preventive measure) (**Table 3**). The IPCITDP also generates a large number of textual features in the form of keywords while processing the textual media stories. These keywords can be used to detect newly emerging epidemiological trends in product-related child injury (**Figure 5**).

**Table 3:** Inclusion of 37 structured variables about product-related child injury between the IPCITDP and the NISS for product-related injury in China

Variable	IPCITDP	Chinese NISS
**Basic characteristics of injury event**		
1. Date the injury event occurred	√	√
2. Geographic location where the injury event occurred	√	
3. Type of place where the injury event occurred	√	√
4. Nature (type) of injury	√	√
5. Body part injured	√	√
6. External cause of injury	√	√
7. Severity of injury		√
8. Clinical diagnosis of injury	√	√
9. Injury outcome (including number of deaths & injured persons)	√	
**Product-related variable**		
10. Number of involved products	√	√
11. Product name	√	√
12. Brand name of product	√	√
13. Characteristics of product	√	
14. Type of product	√	√
15. Disposition of injury	√	√
**Children and supervisor-related variable**		
16. Number of involved children	√	
17. Age of injured child	√	√
18. Sex of injured child	√	√
19. Name of child		√
20. ID number of child		√
21. Registered permanent residence of child		√
22. Education degree of child		√
23. Job of child		√
24. Telephone number		√
25. Activity of child(ren) as injury happened	√	√
26. Number of supervisors	√	
27. Age of supervisor	√	
28. Sex of supervisor	√	
29. Supervisor of the injured child	√	
30. Whether the supervisor was physically with the child when the injury event occurred	√	
**Other variables related to the injury environment and causal factors**		
31. Human-related etiological factor	√	
32. Product-related etiological factor	√	√
33. Weather conditions	√	
34. Air quality	√	
35. Temperature	√	
36. Other adverse environmental conditions	√	
37. Product-related preventive measure	√	

IPCITDP – Internet-based Product-related Child Injury Textual Data Platform, NISS – National Injury Surveillance System

### Early detection of new product-related child injury characteristics

**Figure 6:**
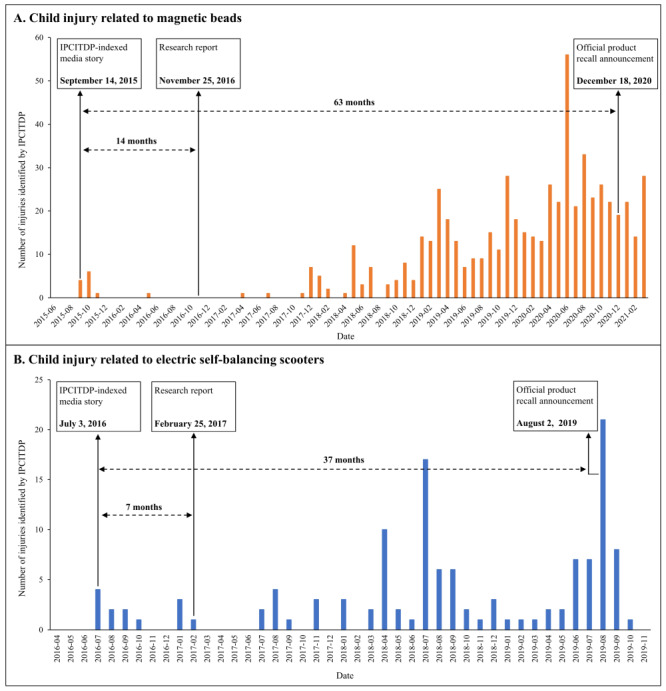
The first detection dates in IPCITDP-indexed media stories, the research literature, and the official product recall announcement for child injury related to magnetic beads and electric self-balancing scooters. **Panel A.** Child injury related to magnetic beads. **Panel B.** Child injury related to electric self-balancing scooters. The first reporting date by research literature was determined by systematic literature search. The first official product recall announcement date was from the official website of the Defective Product Administrative Center (DPAC) of the State Administration for Market Regulation of the People’s Republic of China. IPCITDP – Internet-based Product-related Child Injury Textual Data Platform.

As case studies, we considered two examples of products that appeared to create new and emerging risk of child injury, magnetic beads and electric self-balancing scooters. These cases demonstrate the potential of IPCITDP for early detection of future and emerging product-related child injury risks. The data from IPCITDP first identified risk of magnetic beads-related child injury on 14 September 2015 and detected 10 relevant injury cases in the two subsequent months, far earlier than the first publication dates about the concern in the research literature on 25 November 2016 [35], or the official recall announcement by the Defective Product Administrative Center (DPAC) of the State Administration for Market Regulation of the People’s Republic of China on 18 December 2020 [36] (**Figure 6**, panel A). Corresponding dates for electric self-balancing scooters risks were 3 July 2016, 25 February 2017 [37], and 2 August 2019 [38] (**Figure 6**, panel B).

**Figure 7:**
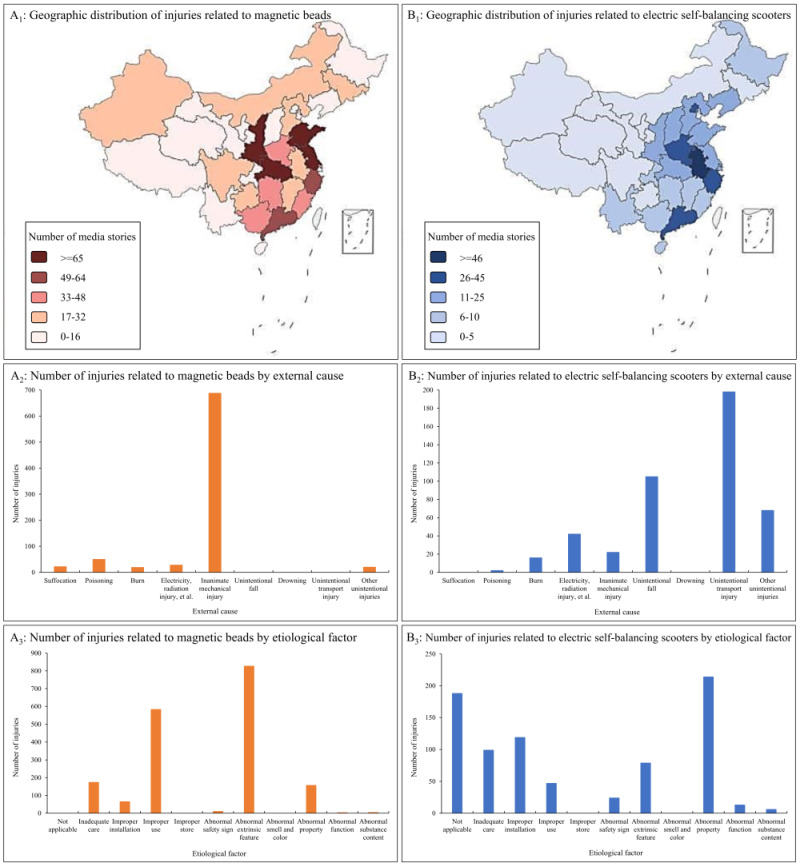
Two examples of potential implications of using IPCITDP to detect newly emerging product-related child injury event risks, 2010–2023. **Panel A.** Data concerning child injury related to magnetic beads. **Panel B.** Data concerning child injury related to electric self-balancing scooters. The IPCITDP collected product-related child injury from media stories of 31 provinces in mainland China, and excluded those from Hong Kong, Macao and Taiwan due different regulations for product-related child injury. IPCITDP – Internet-based Product-related Child Injury Textual Data Platform.

Once the IPCITDP identifies emerging trends in product-related child injury, it can be used to display characteristics of those injuries by geography, external cause, or etiological factors of product-related child injury. **Figure 7**, panels A–B, presents examples related to the two newly emerging product risks, magnetic beads and electric self-balancing scooters. As shown in the figure, patterns such as geographic variations, external cause, and etiological factors of the two new types of product-related injuries emerged clearly from analysis of IPCITDP data.

## DISCUSSION

### Development of the IPCITDP

The IPCITDP was successfully designed as the first data platform that automatically and systematically collects product-related child injury media stories from across mainland China, transferring the data for use to identify emerging injuries and implement prevention strategies. The IPCITDP was developed through four rigorous steps: (1) determining relevant media sources; developing search strategies, text-processing criteria, and a keyword dictionary; formulating the web crawler algorithm according to injury prevention theories, national laws and regulations and relevant literature [3,23–29]; and multiple research group discussions; (2) developing the media story classification model and information extraction algorithms for 29 structured variables based on the prior research [29]; (3) developing a structured query language (SQL) database to dynamically store relevant data [33]; and (4) designing the data platform. Specific discussion about the performance and limitations of IPCITDP appears below.

### Performance of the IPCITDP

#### Classification model and information extraction algorithms

The BERT data classification model we developed performed excellently in identifying eligible media stories on product-related child injury (accuracy = 0.9703), and superior to the performance of BERT-based classifiers for other medical and health patterns (e.g. accuracy of 0.9271 and 0.8615, respectively, in classifying media stories on road traffic crashes [29] and a medical natural language inference data set [39]).

The accuracy of the information extraction algorithms was over 70% for 25 of the 29 structured variables, which was acceptable compared to other product-related online media data mining research projects [40,41]. However, the algorithms did perform poorly for the four remaining variables, with accuracy between 3.23 and 24.76%. The low accuracy for those variables might be primarily a result of two factors. First, journalists may prepare reports with bias or negligence, often unintentionally [42]. Nearly all domestic journalists work without receiving standard training concerning the reporting of injury events [43,44], and many social media stories are prepared by individuals without any journalism training. Thus, media stories may be influenced by the subjective understanding of journalists and other authors. Second, reports may be limited by the publications’ word count limits and thus omit relevant details of injury events. This is particularly true of social media stories, which are typically brief in length and increasingly popular among consumers in recent years [45,46].

Despite the acceptable performance for most variables, our classification model and information extraction algorithms might be less accurate when the injury events are reported in atypical manners, like some social media news stories, and when novel words or injury terms appear. Therefore, it is necessary for administrators to regularly include more training data to update the classification model and information extraction algorithms.

#### Broad geographic coverage

The IPCITDP system achieved geographic coverage of nearly 100% of China, both based on the location of media sources and the location of product-related child injury events. This suggests indexing was thorough and IPCITDP data are unlikely to omit relevant media stories in China. The absence of media stories for nine of 333 prefecture-level administrative divisions (3%) might be explained by the small population in those areas as well as underdeveloped traditional and social media sources there [29]. For example, the three sparsely populated western provinces in China, Tibet, Qinghai and Ningxia, have just six, 17, and 12 licensed internet news media sources, respectively, and 25, 89, and 84 government-permitted social media sources. These figures represent the lowest ranks among the 31 Chinese provinces [21]. According to the national statistics, the populations of Tibet, Qinghai, and Ningxia were 3.64, 5.95, and 7.28 million, respectively, at the end of 2022 [47]. The scarcity of news stories may underestimate the severity of product-related injury in those sparsely populated regions; alternatively, the small population is simply less likely to be injured through exposure opportunity. Future research is recommended to determine whether the IPCITDP system may overlook newly emerging injury characteristics in these sparely-populated regions of China.

#### Provision of supplementary data

The IPCITDP supplements other existing data sources, including the National Injury Surveillance System of China, in two key ways.

First, it collects critical data concerning key variables such as injury outcomes of children and supervisors, characteristics of the product, and human-related etiological factors of the injury event, which are lacking in other data sources and offer rich data to deepen our understanding of the epidemiological characteristics of product-related child injury.

Second, the IPCITDP generates keywords while pre-processing the media stories. As of 31 December 2023, over 200 000 specific keywords were identified and stored by the IPCITDP (**Figure 5**). The still-accumulating database offers remarkable utility to detect newly emerging characteristics of product-related child injury and to refine or expand operational details of the 29 structured variables defined by the IPCITDP. **Figure 6** and **Figure 7** demonstrate the advantages of IPCITDP data in detecting two newly emerging product-related child injuries, thus supplementing information available from the National Injury Surveillance System of China.

#### Early detection of new epidemiological characteristics

**Figure 6** illustrates the advantage of IPCITDP to assist with early detection of new and emerging product-related child injury risks over existing strategies such as the research literature or official government product recall announcements. Through IPCITDP, we can detect early signals about products creating child injury risk, monitor trends and investigate factors creating those trends, and then take decisive and efficient action to reduce injury burden when needed. Specifically, when product defects are reported by the media, the appropriate government department can validate the accuracy of those reports, evaluate the product’s safety, and either recall the products or restrict their sale if the products are determined to be dangerous. For example, the government could have investigated and then recalled the defective magnetic beads when multiple injury stories were detected in media reports after the initial media report published on 14 September 2015, much earlier than the official recall announcement on 18 December 2020 [36]. Such action would likely have prevented many serious injuries and deaths among Chinese children.

#### Data privacy, security, and ethical considerations

The data collected on the IPCITDP platform are published on media websites and social media platforms that are certified by Chinese Cybersecurity Law to publish media stories [25]. Thus, our data involve no illegal information and do not violate individual privacy. All data from the IPCITDP platform will be used for scientific research, government and industry decision-making, and public health practice for injury prevention.

### Limitations of the IPCITDP

Despite its many advantages, the IPCITDP has limitations also.

First, IPCITDP data cannot be directly applied for statistical inference including parameter estimation and hypothesis testing, since they are based on selective reports by the media. In other words, there may be selection bias present from the writer and editor of the news source [48,49]. Further, bias may emerge through the simple act of journalistic reporting. The data from IPCITDP rely on media stories and are therefore inevitably affected by the quality of the authors’ reports, which vary widely. When expert evaluations for product-related injury events are unavailable, the writer of the article may rely on reports from people involved in the injury event, such as the child victim, the child’s parents, or spectators. These reports may be biased, and the author’s interpretation could lead some media-reported information to fall short of desired scientific rigor (e.g. classification of product, cause of injury). Thus, IPCITDP should supplement but not replace official statistics, such as estimates based on national or local surveillance systems.

Second, IPCITDP data are sparse in thinly-populated areas of Western China, including Tibet (n = 14) and Qinghai (n = 166). Data were missing for nine prefecture-level administrative divisions. The lack of data from these regions is likely due to a combination of low population density and underdeveloped media. It may lead to underestimation of the severity of product-related injury or overlook unique injury characteristics from these regions, although a large portion of commercial products are sold nationally or regionally in China, minimising concerns from this limitation somewhat. We do recommend, however, that the IPCITDP data to be used to identify early and emerging characteristics of product-related injury and then be merged with other data sources to guide injury prevention decision-making and practice by governmental departments.

Third, most media stories collected by the IPCITDP included only information about key variables. Missing in many cases were details such as the product manufacturer and the model, size, and batch number of the product.

Last, the IPCITDP started to collect data prospectively on 18 November 2020, and therefore cannot retrospectively search all eligible media stories because many reports are no longer available online. Most media sources cannot afford the expensive storage cost of rapidly increasing media stories and thus remove media stories published in early years from their official websites [50].

### Next steps

We suggest several optimisations and extensions to improve the IPCITDP or similar systems in the future.

(1) Continuously refine and improve search strategies, the keyword dictionary, and filtering criteria, as well as the 29 structured variables, to include new topics and stories relevant to product-related child injury events and to identify newly emerging product-related child injuries.

(2) Improve the accuracy of the media story classification model and information extraction algorithms, especially for several structured variables with low information extraction accuracies; this can be accomplished through increasing the number of manually labelled media stories and improving classification model and information extraction algorithms.

(3) Explore statistical models to support inference testing using non-probability sample data and predictive analytics, which IPCITDP data represent, to estimate population product-related child injury statistics, monitor early warning signals, and assess the effectiveness of prevention programmes. In doing so, the inconsistent development of the media industry across geographic locations (province and prefecture-level administrative division) should be considered, especially when estimating provincial or prefectural-level product-related injury patterns.

(4) Link IPCITDP data with medical records and official injury surveillance records, and incorporate interdisciplinary approaches including psychological science, engineering dynamics, and product design into assessment and prevention strategies. This strategy enables full use of available data sources to best serve the prevention of product-related child injury.

(5) Expand the target population of product-related injury data platform from children to all age groups, generalise the approach to other major injury causes like falls, and improve the user interface design of the data platform to better satisfy the needs of the public and key stakeholders.

## CONCLUSIONS

We developed the IPCITDP online platform to automatically acquire, process, and store data concerning media-reported product-related child injuries in the Chinese language every 24 hours. The IPCITDP supplements existing data sources, including the national official injury surveillance system of China, to collect relevant and real-time product-related child injury data. The IPCITDP can be used in multiple productive ways, including to identify poorly-understood epidemiological characteristics of product-related child injury and to provide early detection of newly emerging epidemiological characteristics of product-related child injury.

## Additional material


Online Supplementary Document
