# Supplementary material for: Development of an Internet-based Product-related Child Injury Textual Data Platform (IPCITDP) in China
Source: J Glob Health. 2024 Aug 23;14:04174. doi: 10.7189/jogh.14.04174 (PMC11342019; doi:10.7189/jogh.14.04174)
Supplement: Online Supplementary Document [file jogh-14-04174-s001.pdf]

Online Supplementary Document

Textbox S1. Criteria for product-related child injury document searching and filtering

Searching strategies

1. Media stories were published in Chinese language.
2. The injury happened between January 1, 2010 and December 31, 2023.
3. The story appeared on an included news media website or social media platforms.
4. The scope of the media story was the full text.
5. Search terms were the combination of "child" AND ("injury" OR "death") in Chinese, plus their synonyms.

Inclusion criteria

1. Media stories were related to product-related child injury, involving the basic definitions and key elements of product-related child injury.
2. The injury happened between January 1, 2010 and December 31, 2023.
3. The injured included children aged 0-14, or the story addressed risk for children because they used the product.
4. Media stories were published in Chinese language.
5. The injury event happened in mainland China.

Exclusion criteria

1. Media stories were related to product-related child injury, but the main content was not focused on the injury event itself. Instances include summary data reports, survey reports, product-related advertisements, risk warnings and measures.
2. Media stories only contained pictures, audio, or video, and lacked any text description.
3. Media stories were related to fictional product-related child injury from games, television dramas, movies, cartoons, or other cultural or commercial works.
4. Media stories were related to psychological injuries rather than physical injuries, or included colloquial or slang expressions that misleadingly implied child injury or death.
5. Media stories appeared with URL link failure, garbled characters, abnormal text displays, or we were otherwise unable to extract relevant text content.

Table S1. Description of 29 variables relevant to product-related child injury (text features)

Variable	Description
**Basic characteristics of injury event**	
1. Date the injury event occurred	Standardized format: year (YYYY)-month (MM)-day (DD)
2. Geographic location where the injury event occurred	Standardized format: province-city-district/county
3. Type of place where the injury event occurred	Categorical variable (8 categories, e.g., home)
4. Nature (type) of injury	Categorical variable (8 categories, e.g., fracture)
5. Body part injured	Categorical variable (7 categories, e.g., head injury)
6. External cause of injury	a) Definition: only unintentional causes were included, excluding the categories like intentional, interpersonal, self-harm, war, and injuries irrelevant to product-related child injury, such as overwork, medical injury and surgical complications.<br>b) Categorical variable (9 categories, e.g., suffocation)
7. Clinical diagnosis of injury	Definition: professional medical diagnosis made by doctor or other health professionals.
8. Injury outcome	a) Definition: the number of injured and killed children and adults.<br>b) Standardized format: number of deaths & injured persons
**Product-related variable**	
9. Number of involved products	Categorical variable (3 categories)
10. Product name	
11. Brand name of product	
12. Characteristics of product	Definition: size, shape, number, and other identifying characteristics of the product.
13. Type of product	Categorical variable (11 categories, e.g., toy)
14. Disposition of injury	Categorical variable (2 categories)
**Children and supervisor-related variable**	
15. Number of involved children	Categorical variable (3 categories)
16. Age of injured child	Standardized format: year/month/day, and convert to year
17. Sex of injured child	Categorical variable (3 categories)
18. Activity of child(ren) as injury happened	Categorical variable (7 categories, e.g., sports)
19. Number of supervisors	Categorical variable (3 categories)
20. Age of supervisor	Standardized format: years old
21. Sex of supervisor	Categorical variable (3 categories)
22. Supervisor of the injured child	Categorical variable (6 categories, e.g., father)
23. Whether the supervisor was physically with the child when the injury event occurred	Categorical variable (2 categories)
**Other variables related to the injury environment and causal factors**	

24. Etiological factors leading to injury	Categorical variable (2 categories: 5 human-related causes and 6 product-related causes)
25. Weather conditions	Categorical variable (5 categories, e.g., sunny day)
26. Air quality	Categorical variable (4 categories)
27. Temperature	Categorical variable (2 categories)
28. Other adverse environmental conditions	Categorical variable (4 categories)
29. Product-related preventive measure	Definition: preventive measures, suggestions, safety warnings related to the injury event.

Table S2. Parameter configuration of the BERT classification model

**Parameter**	**Value**
Pre-training model	Chinese_L-12_H-768_A-12
Application software	Python3.7
System framework	Tensorflow2.2
Number of human-annotated samples	
Total	10,000
Train set	8,000 (80%)
Validation set	1,000 (10%)
Test set	1,000 (10%)
Optimizer	Adam
Maxlen	500
Epoch	18
Learning rate	1e-6
Batch size	8
Method to prevent overfitting	Dropout
Method to callback	a) Early Stopping;<br>b) Model Check Point;<br>c) Reduce LR on Plateau
Indicator of reducing learning rate	
Monitor	val_loss
Mode	auto
Factor	0.1
Patience	2

BERT – Bidirectional Encoder Representations from Transformers.

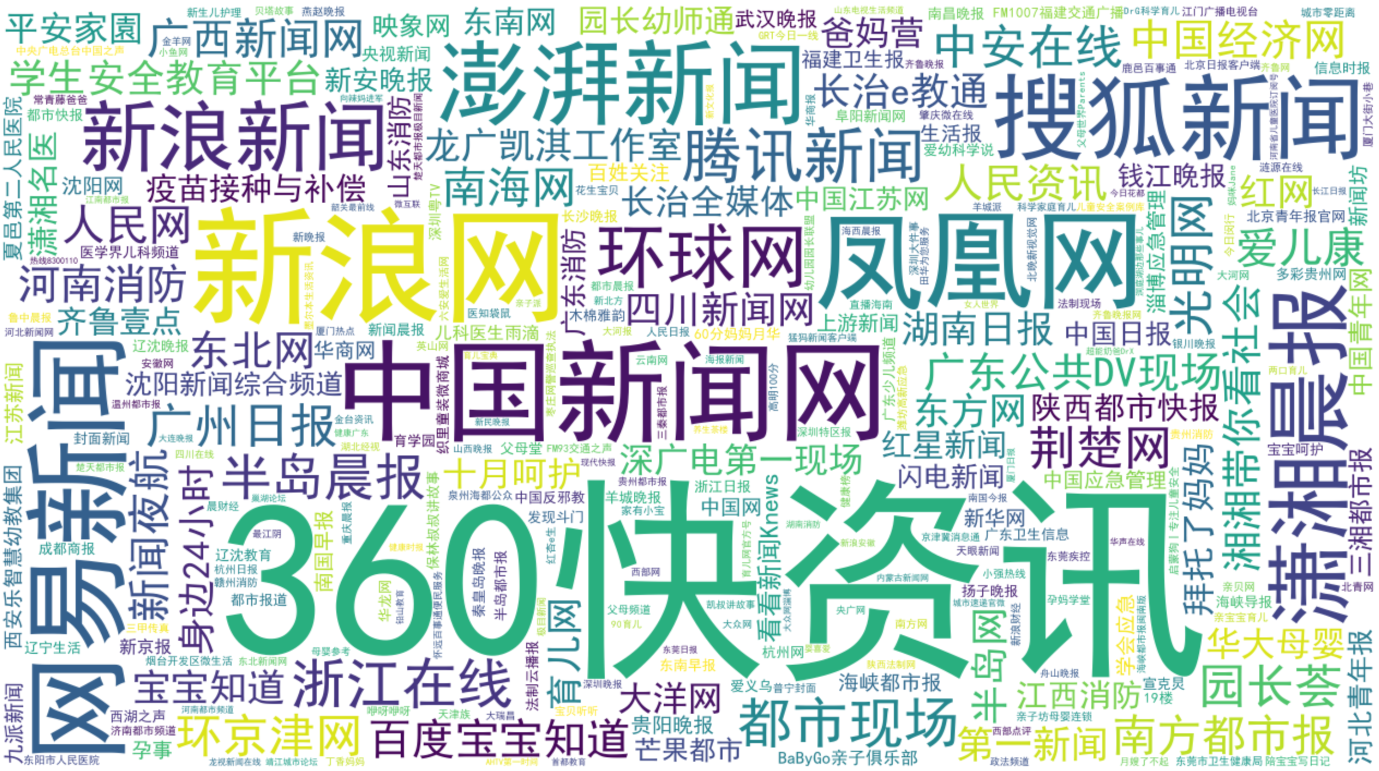

Figure S1. Word cloud of platform-involved news media websites or social media accounts reporting product-related child injury in mainland China, as of December 31, 2023. As of December 31, 2023, a total of 13,261 news media websites or social media accounts reported product-related child injury and were included in the data platform. This word cloud is based on data from the 300 news media websites and social media accounts that most frequently reported product-related child injury. Larger Chinese characters indicate that the media reported more stories about that topic in relation to product-related child injury.
